# Technical variation of trans-articular sacroiliac joint (SIJ) fusion using three screws considering the effects of sacral dysplasia in patients with non-traumatic SIJ pain

**DOI:** 10.1186/s12891-019-2771-1

**Published:** 2019-08-28

**Authors:** Soo-An Park, Dai-Soon Kwak, Ho-Jung Cho

**Affiliations:** 1 Department of Orthopedic Surgery, Chung General Hospital, 76 Sujeong-ro, Sujeong-gu, Seongnam-si, Gyeonggi-do 13316 South Korea; 2 Catholic Institute of Applied Anatomy / Department of Anatomy, College of Medicine, The Catholic University of Korea, 222 Banpo-daero, Seocho-gu, Seoul, 06591 South Korea

**Keywords:** Sacroiliac joint, Non-traumatic SIJ pain, Trans-articular sacroiliac joint fusion, Sacral dysplasia

## Abstract

**Background:**

This study evaluated the technical adequacy of trans-articular sacroiliac joint (SIJ) fusion using three screws for non-traumatic SIJ pain, considering different grades of sacral dysplasia.

**Methods:**

Cadaveric CT data of unilateral sacropelvic complexes for 72 individuals (53.4 ± 8.4 years) were selected. A 3D model was reformatted into the plain lateral radiograph to mark the articular surface of the SIJ. Subjects were classified into dysplastic (DYS) and non-dysplastic sacrum (NDS) groups. Proximal (PS), middle (MS), and distal screws (DS) with 10-mm diameter were virtually introduced to the iliac bone and the SIJ on the lateral image with a 5-mm safety margin. On a corresponding axial image, each screw was advanced vertically to the sagittal plane with the same safety margin. The entry points for each screw to the endplate of S1 (S2) and to the corresponding anterior sacral margin on the lateral image were measured, along with the maximal screw lengths on the axial image. Whether each screw passed through the SIJ was determined. Different types of sacral dysplasia and screws were compared statistically.

**Results:**

Thirty-eight (26.4%) cases were DYS, and 106 (73.6%) were NDS. The entry points of all screws were significantly more distal in DYS than in NDS groups. The PS and MS screw lengths differed significantly between the 2 groups. Incidences of short sacral fixation (< 10 mm) were significantly higher for the DS in both NDS (38.7%) and DYS (39.5%) groups. Incidences of screw pass were lowest for the MS in both NDS (43.4%) and DYS (47.4%) groups.

**Conclusions:**

Sacral dysplasia locates the SIJ more distally and therefore affects the entry point locations and screw lengths for all screws in trans-articular SIJ fusion, compared with a non-dysplastic sacrum. Moreover, three-screw fixation risks the development of unstable DS fixation and a high extra-articular fixation rate in MS.

## Background

Pain from the sacroiliac joint (SIJ) is mostly in the buttock, lower back, and inguinal region and is similar to pain from the lumbosacral region [1–3]. The incidence of SIJ pain accounts for 15–30% of total lower back pain cases [1, 4]. Infectious, spondyloarthropathic, or degenerative sacroiliitis and traumatic spondylopelvic dissociation are frequent causes of SIJ pain [5, 6]. Surgical sacroiliac stabilization for SIJ fusion can be performed for patients with persistently unresponsive SIJ pain after non-surgical treatment [7–9] and after documenting evidence of SIJ as a source of pain. Diagnosis of SIJ pain warranting fusion should be based on pain at or close to the posterior superior iliac spine, positive findings on multiple provocative physical examinations and pain reduction on fluoroscopically-guided local anesthetic injection into the SIJ [1].

Trans-iliosacral fixation is performed in patients with traumatic sacroiliac dissociation using two long screws unilaterally or bilaterally to pass the midline of the sacrum [10, 11]. During trans-iliosacral fixation, the screw can injure adjacent neurovascular structures by violating the sacral bony cortex. This type of iatrogenic injury occurs frequently in patients with sacral dysplasia. Sacral dysplasia is a dysmorphic upper sacrum that causes frequent misplacement of trans-iliosacral screw fixation in surgeries for traumatic sacropelvic dissociation [10, 12, 13].

Trans-iliosacral fixation techniques for non-traumatic SIJ pain differ from those for traumatic cases by using trans-articular fixation for SIJ fusion, mostly unilateral fixation, and two or three shorter screws [14–17]. However, there is a lack of guidelines for trans-articular fixation for non-traumatic SIJ pain, and even more technical variation occurs and requires guidelines when considering sacral dysplasia.

The objectives of this study were to establish a proper technique for percutaneous trans-articular iliosacral fixation for SIJ fusion and to define the clinical implications of sacral dysplasia for non-traumatic SIJ pain by determining the entry point and maximal screw length available when using 3 screws with maximally available screw diameter and conventional trajectory and maintaining 5 mm-safety margins to the adjacent sacral cortex in the presence of sacral dysplasia.

## Methods

Cadaveric CT data for 144 unilateral sacropelvic complexes from 72 individuals (34 males and 38 females; mean age, 53.4 ± 8.4 years) were selected from the registration of the Catholic digital human library; the chosen subjects had no deformities, fractures, or surgeries to the lumbo-sacropelvic bones [18, 19]. The slice thickness for CT scanning of the cadaveric body in this study was between 0.625 mm and 1.0 mm, and the pixel dimensions of CT images were between 0.625 mm and 0.832 mm.

### Image processing

**Fig. 1:**
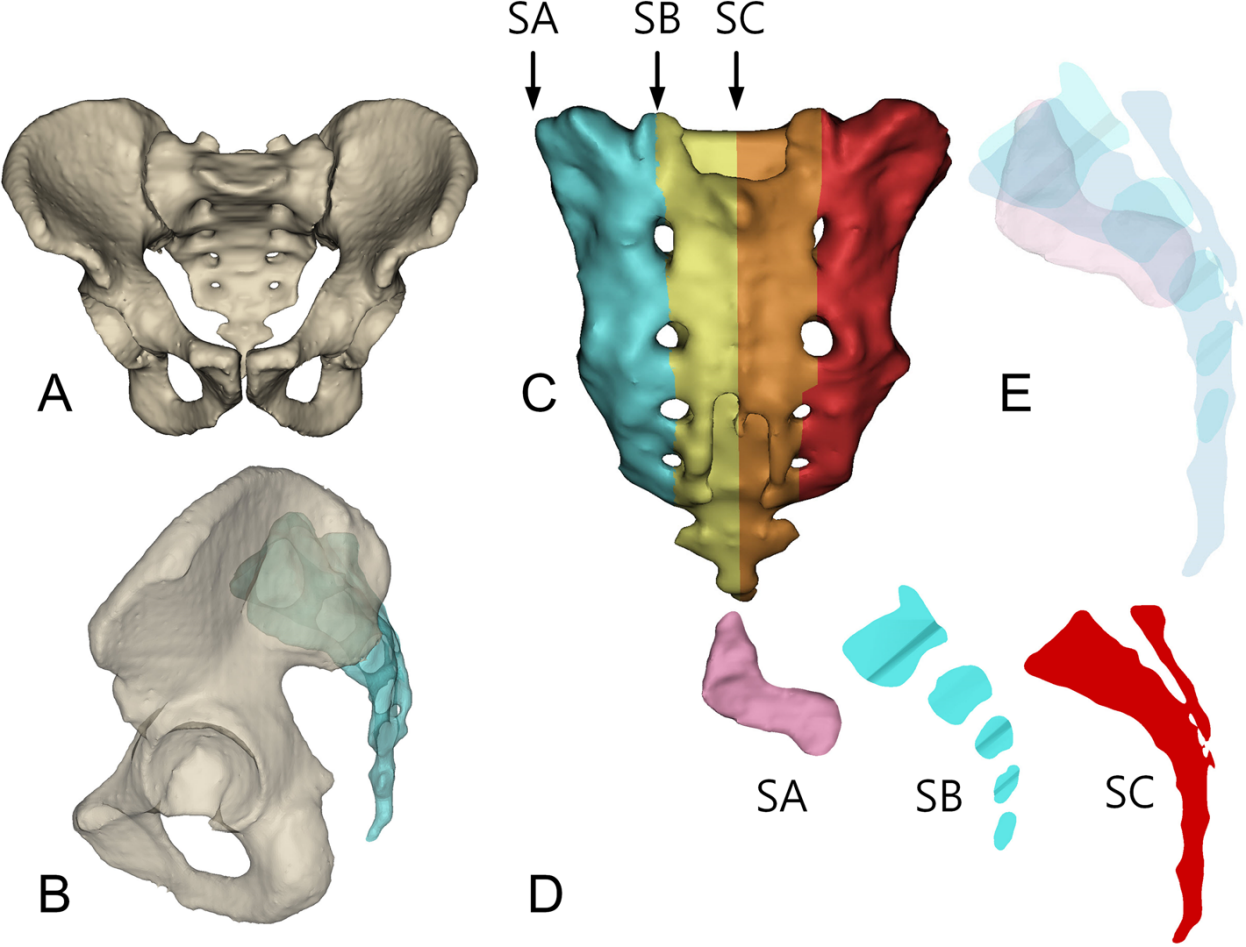
Procedures for image processing: (**a**) 3D reconstruction of sacropelvic complex; (**b**) transparently reconstructed 2D lateral view, but clearly delineating the sacral canal and foramens and the articulating margin of the SIJ is difficult; (**c**) 3 section views marked on the 3D image of a sacrum; (**d**) 3 section views with the articular surface of the SIJ (SA), the sagittal trans-foraminal image (SB), and the mid-sagittal image of a sacrum (SC); (**e**) a sagittal 2D image of a sacrum was reconstructed for the unilateral sacropelvic complex by combining three transparent section views

A 3D model of each sacropelvic bone complex was reconstructed from the CT data using the Mimics (18R, Belgium) software [18, 20]. In the software, a sagittal image of the sacrum for the unilateral sacropelvic complex was made by combining three transparent images of the sacral articular surface of the SIJ, the sagittal trans-foraminal image to connect the centers of adjacent sacral foramens by the vertical and step-down lines from the corresponding upper endplate of S1 to the sacral end, and the mid-sagittal image bisecting the sacrum (Fig. 1).

### Virtual trans-articular iliosacral fixation

**Fig. 2:**
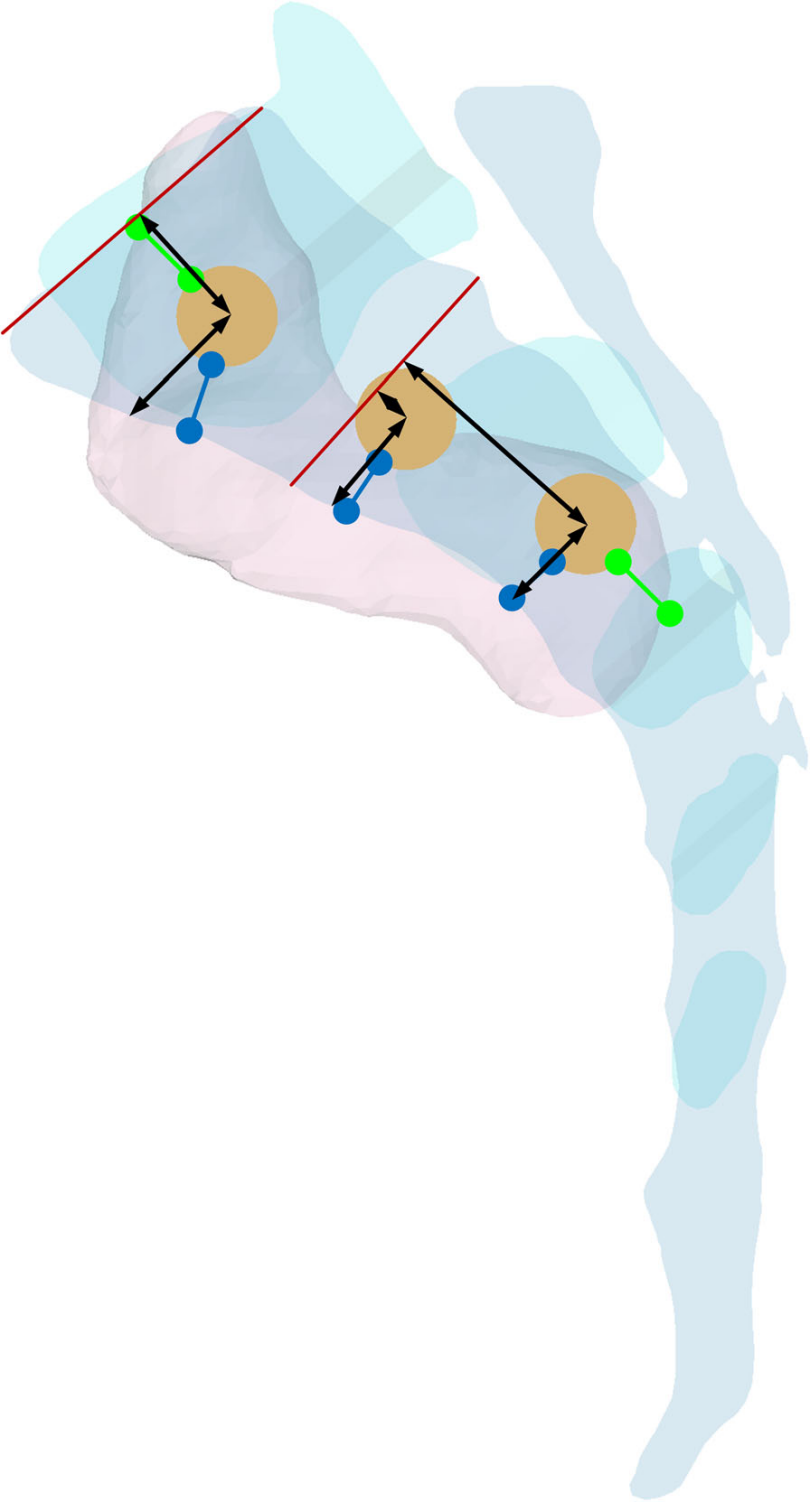
Entry points for the proximal-to-distal and anterior-to-posterior distances for three screws. The safety margin from each screw to the margin of the SIJ is marked with green lines, and that to the margin of the corresponding anterior margin of the sacrum is marked with blue lines

In the software, three virtual screws were made with 10-mm cylinders and applied to the reconstructed sagittal images [5, 21]. The proximal screw (PS) was applied with the least safety margin (5-mm) from the proximal to the anterior margin of the sacroiliac articular surface and from the anterior margin of the mid-sagittal image [22]. The distal screw (DS) was applied with a same safety margin from the distal to anterior margin of the sacroiliac articular surface and from the anterior margin of the mid-sagittal image. The middle screw (MS) was applied at the center between the PS and DS, also maintaining a same safety margin from the anterior margins of the sacroiliac articular surface and the mid-sagittal image (Fig. 2).

**Fig. 3:**
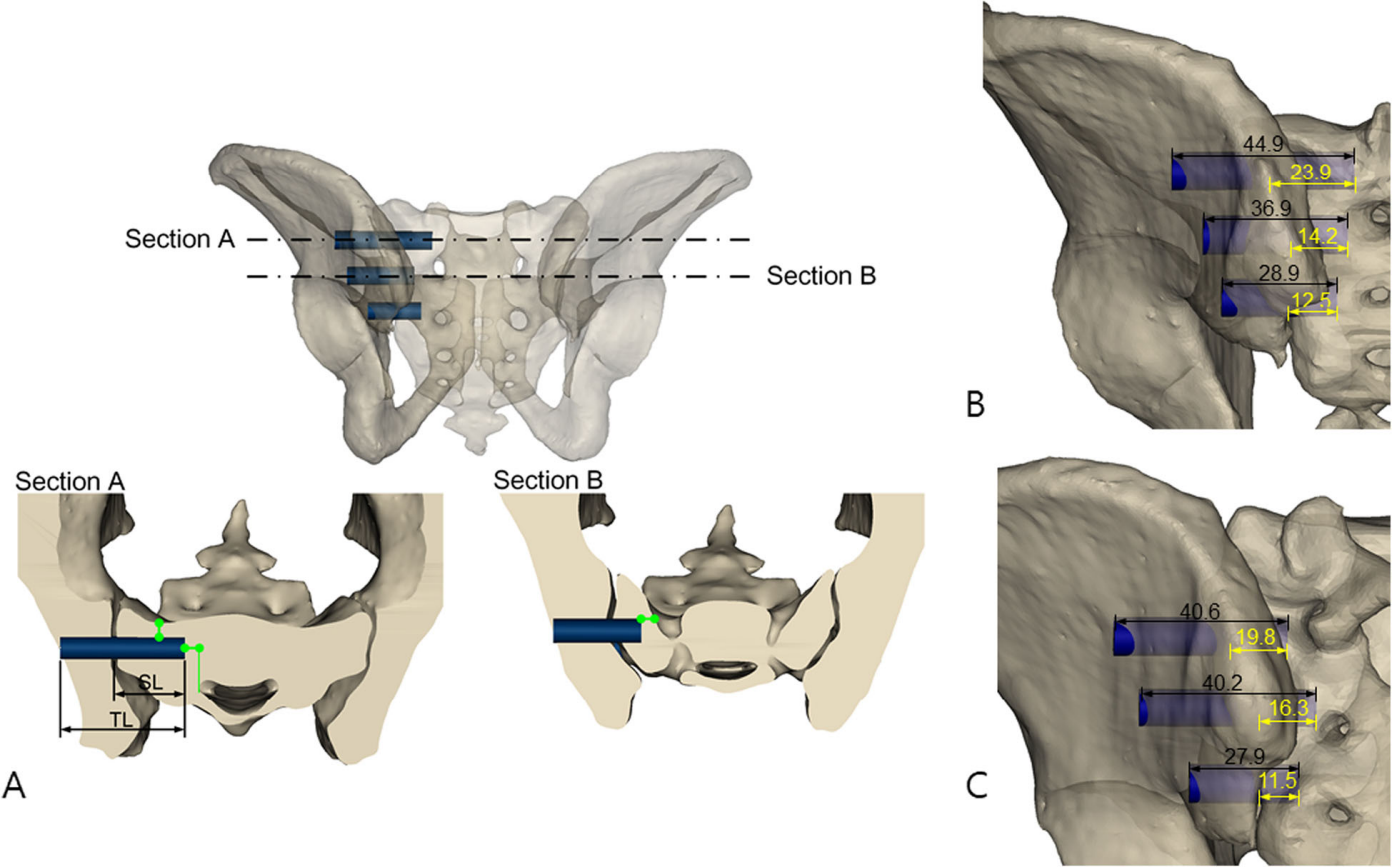
Maximal screw length (**a**) for the iliac and sacral bones (total screw length, TL) and just the sacrum (sacral screw length, SL). Section A indicates the proximal screw fixation, and section B indicates the middle screw fixation. Green lines indicate the safety margins to the lateral margin of the sacral canal and the anterior margin of the sacrum and foramen. Screws with certain length (within mean maximal length) used in (**b**) non-dysplastic sacrum (**c**) dysplastic sacrum

After establishing the entry points on the sagittal image, each screw was inserted vertically from the lateral margin of the iliac bone to the mid-sagittal plane on the corresponding axial image and stopped at the point required to maintain the 5-mm safety margin from the lateral margin of the canal without violating the margins of the anterior sacrum, canal, or foramens (Fig. 3).

### Parameters

The entry point of each screw was measured on the sagittal image. For the PS, the entry point for the proximal-to-distal distance was measured as the shortest distance from the center of the PS to the upper endplate of S1. For the MS and DS, the entry points for the proximal-to-distal distance were measured from the center of each screw to the upper endplate of S2. The entry point for the anterior-to-posterior distance for the PS was measured as the distance from the center of the screw to the anterior margin of the mid-sagittal image on the line parallel to the upper endplate of S1. For the MS and DS, the entry point for the anterior-to-posterior distance was measured as the distance from the center of each screw to the anterior margin of the mid-sagittal image on the line parallel to the upper endplate of S2 (Fig. 2).

The maximal length of each screw was measured on the axial image. The maximal lengths of the three screws passing the iliac and sacral bones (total screw length) and that passing just the sacrum (sacral screw length) were measured on the corresponding axial image (Fig. 3). Both authors measured the entry point and screw length to see how well the two values matched for each parameter, but data from just one observer were used for further analysis.

Whether the screw passed through the SIJ was determined by the percentage of the screw circle occupying the articular surface on the axial image. If the screw circle occupied > 50%, it was categorized as *pass*. If the screw circle occupied ≤50%, it was categorized as *no-pass*.

### Dysplastic and non-dysplastic sacrums

**Fig. 4:**
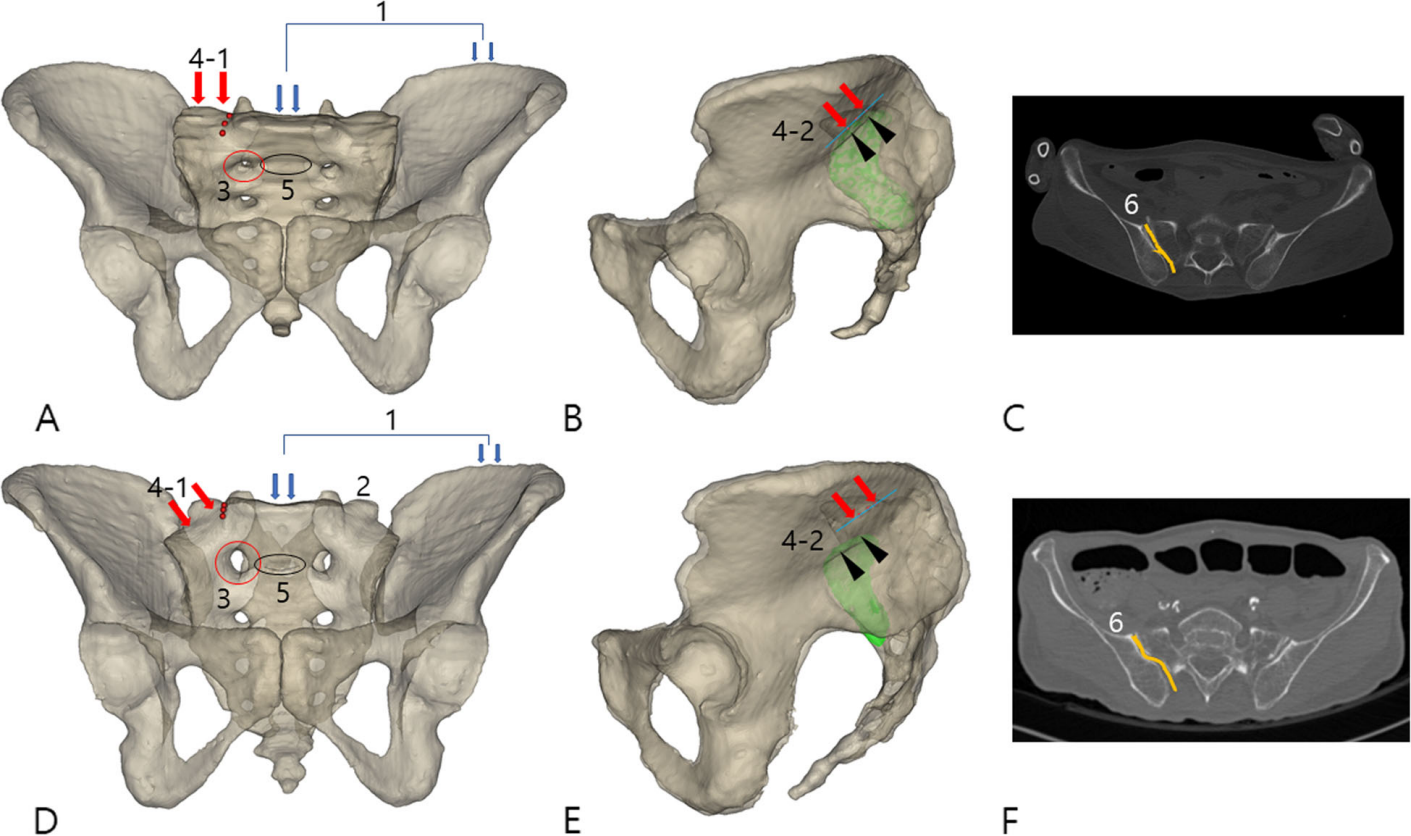
Non-dysplastic sacrum (**a**) pelvic outlet image; (**b**) lateral image of sacropelvic complex, including the articular surface of the SIJ (green) and the iliac cortical density (ICD, black arrowhead); (**c**) axial CT image of the sacropelvic complex; and dysplastic sacrum (**d**) pelvic outlet image; (**e**) lateral image; (**f**) axial CT image. The differences between the non-dysplastic and dysplastic sacra are described in Table 7

Using a previously published guideline [10], a dysplastic sacrum was defined using the amount of S1 body prominence over the iliac cortical density (ICD) on the reconstructed sagittal image. Sacrums were categorized as non-dysplastic (NDS) and dysplastic (DYS) when they had less and more than 50% of the S1 body proximal to the ICD, respectively (Fig. 4).

### Statistical analyses

To compare demographic data between the two-sacrum (NDS and DYS) groups, a student t-test was performed for age, and a chi-square test was performed for the sex ratio (Table 1).

The entry points of the proximal-to-distal and anterior-to-posterior distances for each screw were compared between the groups using one-way ANOVA, with the entry point as the dependent variable and the sacrum group as an independent variable (Table 2).

One-way ANOVA were also performed to compare the total and sacral screw lengths between the groups, with the screw length as the dependent variable and the sacrum group as an independent variable. Within each group, the total and sacral screw lengths between screws (PS, MS, and DS) were compared by one-way ANOVA, with the screw length as the dependent variable and the screw as an independent variable. When significance was found on the screw variable, post-hoc analyses were used to analyze the differences between the screws (Tables 3 and 4). A reliability analysis was performed using two values and two observers for each parameter.

Chi-square tests were performed to compare the incidence of short (< 10 mm) sacral screw length (Table 5) and screw passage through the articular surface of the SIJ (Table 6) between the groups and among the three screws within each group.

## Results

The NDS and DYS groups contained 106 (73.6%) and 38 (26.4%) unilateral sacropelvic complexes, respectively. The age of the two groups did not differ significantly. However, the sex ratio of the two groups did differ significantly: the NDS group contained more females, and the DYS group contained more males (Table 1).

**Table 1:** Demographics

	NDS	DYS	P
N	106 (73.6%)	38 (26.4%)	
Age (years)	54.0 ± 8.3	51.6 ± 8.7	0.138
Sex (N)	44 males (41.5%)	24 males (63.2%)	0.022
	62 females (58.5%)	14 females (36.8%)	

### Entry point

Each entry point for the proximal-to-distal distance of all three screws was significantly more proximal in NDS than in DYS groups. The entry point for the anterior-to-posterior distance of the MS was significantly more posterior in DYS than in NDS groups, and those for the PS and DS did not differ significantly between the groups (Table 2). The entry points measured by the two observers for all three screws in two groups were not significantly different with the pair-wise comparisons, and all parameters demonstrated significant correlations between two observers with the intra-class correlation coefficient (ICC) values from 0.891 to 0.958.

**Table 2:** Entry points of each screw for the proximal-to-distal and anterior-to-posterior distances on the reconstructed sagittal image

	Entry Point (proximal-to-distal)		P
	NDS	DYS	NDS vs. DYS
PS (mm)	14.7 ± 6.8	26.9 ± 4.1	< 0.001
MS (mm)	3.8 ± 8.0	15.9 ± 5.2	< 0.001
DS (mm)	20.9 ± 9.5	32.9 ± 7.0	< 0.001
	Entry Point (anterior-to-posterior)		P
	NDS	DYS	NDS vs. DYS
PS (mm)	12.6 ± 3.3	13.8 ± 4.7	0.097
MS (mm)	10.3 ± 2.2	12.1 ± 5.1	0.004
DS (mm)	10.2 ± 1.2	10.2 ± 0.3	0.855

### Maximal screw length

The total screw length of the PS was significantly longer in NDS (mean, 44.9 mm) than in DYS (40.6 mm) (*P* <  0.001), and that of the MS was significantly longer in DYS (40.2 mm) than in NDS groups (36.9 mm) (P <  0.001). The total screw lengths of the DS did not differ significantly between the groups. The total screw length of the PS, MS, and DS became significantly longer in the NDS group in that order. In the DYS group, the total screw lengths of the PS and MS were significantly longer than that of the DS, but the PS and MS did not differ significantly from each other (Table 3).

**Table 3:** Maximal screw length for the iliac and sacral sides (total screw length)

	NDS		DYS		P
	Mean (SD)	P	Mean (SD)	P	NDS vs. DYS
		PS vs. MS		PS vs. MS	
		MS vs. DS		MS vs. DS	
		DS vs. PS		DS vs. PS	
PS (mm)	44.9 (5.0)	< 0.001	40.6 (4.6)	0.177	< 0.001
MS (mm)	36.9 (3.7)	< 0.001	40.2 (3.1)	< 0.001	< 0.001
DS (mm)	28.9 (6.5)	< 0.001	27.9 (3.8)	< 0.001	0.829

The sacral screw length of the PS was significantly longer in NDS (23.9 mm) than in DYS (19.8 mm) (P <  0.001), and that of the MS was significantly longer in DYS (16.3 mm) than in NDS groups (14.2 mm) (*P* = 0.009). The sacral screw lengths of the DS did not differ significantly between the groups. The sacral screw lengths of the PS, MS and DS became significantly longer in that order in both groups (Table 4).

**Table 4:** Maximal screw length for the sacral side (sacral screw length)

	NDS		DYS		P
	Mean (SD)	P	Mean (SD)	P	NDS vs. DYS
		PS vs. MS		PS vs. MS	
		MS vs. DS		MS vs. DS	
		DS vs. PS		DS vs. PS	
PS (mm)	23.9 (4.4)	< 0.001	19.8 ± 3.5	< 0.001	< 0.001
MS (mm)	14.2 (4.0)	< 0.001	16.3 ± 4.0	< 0.001	0.009
DS (mm)	12.5 (5.3)	< 0.001	11.5 ± 4.2	< 0.001	0.284

The maximal lengths for total and sacral screws measured by the two observers for all screws in two groups were not significantly different with the pair-wise comparisons, and all parameters demonstrated significant correlations between two observers with the ICC from 0.901 to 0.977.

### Incidence of short sacral fixation

Incidences of short sacral fixation (< 10 mm) for the PS, MS, and DS did not differ significantly between the groups. The incidence for the DS [N (%), 41 (38.7%)], MS [12 (11.4%)], and PS [0 (0%)] was significantly higher in that order in NDS group. The incidence for the DS [15 (39.5%)] was significantly higher than that for the PS [0 (0%)] and MS [3 (8.6%)], but the differences between the PS and MS were not significant in DYS group (Table 5).

**Table 5:** Incidence of sacral screw length < 10 mm (short sacral fixation rate)

	NDS		DYS		P
	N (%)	P	N (%)	P	NDS vs. DYS
		PS vs. MS		PS vs. MS	
		MS vs. DS		MS vs. DS	
		DS vs. PS		DS vs. PS	
PS	0 (0)	< 0.001	0 (0)	0.065	NA
MS	12 (11.4)	< 0.001	3 (8.6)	0.002	0.636
DS	41 (38.7)	< 0.001	15 (39.5)	< 0.001	0.931

### Incidence of screw pass through the SIJ

Incidences of screw pass through the articular surface of the SIJ by the PS, MS, and DS did not differ significantly between the groups. The incidence for the MS, DS, and PS became significantly lower in that order in NDS [N (%), 46 (43.4%); 67 (63.2%); 104 (98.1%)] and DYS groups [18 (47.4%); 28 (73.7%); 38 (100%)], respectively (Table 6).

**Table 6:** Incidence of screw pass through the articular surface of the sacroiliac joint (pass ratio)

	NDS		DYS		P
	N (%)	P	N (%)	P	NSD vs. DYS
		PS vs. MS		PS vs. MS	
		MS vs. DS		MS vs. DS	
		DS vs. PS		DS vs. PS	
PS	104 (98.1)	< 0.001	38 (100)	< 0.001	0.394
MS	46 (43.4)	0.004	18 (47.4)	0.019	0.672
DS	67 (63.2)	< 0.001	28 (73.7)	0.001	0.242

Screw pass was determined when the screw passed through > 50% of the articular surface of the SIJ

## Discussion

Trans-articular iliosacral fixation to treat the non-traumatic SIJ pain is to maintain stabilization before obtaining the bony fusion of SIJ, not to securely stabilize the broken SIJ. Therefore, the screw fixation for non-traumatic SIJ pain should be minimally invasive, using shorter screws for safety not violating the adjacent structures, and underwent technically easily under the standard fluoroscopic images. That is why the three-screw fixation using short implants is preferred in fusion of non-traumatic SIJ pain, and the vertical trajectory to the sagittal plane of sacrum is preferred in screw fixation [23].

The entry point of the PS for trans-iliosacral fixation is established using a lateral fluoroscopic image of the sacrum below the ICD at the space between the anterior and posterior margins of the sacral body and under the pelvic outlet view at the space between the endplate and foramen of S1. After establishing its entry point, the PS can be advanced following the fluoroscopic guidance of the pelvic inlet view to monitor the violation of the anterior and posterior margins of the sacral body. In this study, the trajectory of the PS was vertical to the mid-sagittal plane of the sacrum and parallel to the S1 endplate. This type of procedure for PS fixation is quite similar in cases with sacroiliac trauma and non-traumatic SIJ pain when the sacrum is not dysplastic [5, 17, 24–26].

After completing PS fixation, the 2nd screw is attempted toward S2 in cases of traumatic sacroiliac dissociation [13], but toward the distal end of the SIJ in cases of non-traumatic SIJ pain [23, 27]. The entry point of the DS for non-traumatic SIJ pain should be made on the distal articular surface of the SIJ using a lateral fluoroscopic image of the sacrum and with consideration of the space needed to hold the MS. Placement of the DS on the distal articular surface of the SIJ made the DS fixation shallow, keeping the mean sacral screw length to about 12 mm and causing sacral fixation of less than 10 mm in about 40% of cases in both groups.

The MS was inserted at the center between the PS and DS, taking care not to violate the anterior or posterior margin of the sacral body or the anterior margin of the SIJ [17, 26]. As a result, the entry points of all three screws were located more distally in DYS than in NDS groups. The entry points for the anterior-to-posterior distance for the PS and DS did not differ, but that for the MS was located more posteriorly in DYS than in NDS groups. Although the entry points for the anterior-to-posterior distance in the MS differed between the 2 groups, the mean locations for all three screws were 10 to 14 mm from the corresponding anterior margin of the sacrum in both groups.

When the sacrum is dysplastic, the screw to S2 becomes the main screw for trans-iliosacral fixation in cases with sacroiliac trauma. In this traumatic case, the screw to S2 is longer to reach the contralateral end of the sacrum than that to S1. In that case, the entry point and/or the trajectory of the S1 screw should be changed and stop before the sagittal midline of the sacrum [12, 13, 24, 25]. The current findings can be applied in sacroiliac trauma to the proximal screw, but not the distal screw in cases of non-dysplastic sacrum, nor to any screws in cases of dysplastic sacrum.

When performing PS fixation for non-traumatic SIJ pain in cases with sacral dysplasia, the entry point should be made more distally with reference to the level of the ICD than in cases without sacral dysplasia. Because the ICD is recognized as the proximal margin of the SIJ, [10] more distally located entry points for all three screws in DYS compared with NDS groups mean that the SIJ in DYS is more distal than it is in NDS. Therefore, sacral dysplasia can be recognized as a factor that locates the SIJ more distally, compared with a non-dysplastic sacrum.

Differences in the proximal-to-distal location of the SIJ according to the presence of sacral dysplasia also affected the screw length. The total and sacral lengths of the PS were shorter in DYS than in NDS, and those for the MS were shorter in NDS than in DYS groups. The DS was consistently shorter in total and sacral lengths than the PS and MS in both groups. Short screw length in trans-articular fixation for non-traumatic SIJ fusion may be an etiologic factor in the lower fusion rate and increased postoperative implant failure rate. However, the effects of short screw length on SIJ fusion were not investigated in the current study.

Thirty-eight cases of sacral dysplasia (26%) were observed in our study population of 144 subjects. The prevalence of sacral dysplasia in this study is similar to previous studies (30 to 50%); it is not uncommon [13]. Sacral dysplasia has been defined as a dysmorphic upper sacral segment. The morphology of the osseous corridor in sacral dysplasia has been previously described as having five associated signs identifiable on pelvic radiographs and one identifiable on an axial CT. An upper sacral segment not recessed in the pelvis, mamillary bodies, a misshapen sacral foramen, an acute alar slope, residual disc, and tongue-in-groove sacroiliac articulation are characteristic findings indicating sacral dysplasia (Fig. 4 and Table 7) [12, 13, 24, 25].

**Table 7:** Radiographic signs differentiating dysplastic and non-dysplastic sacra

	Dysplastic sacrum	Non-dysplastic sacrum	Identifiable images
1	Upper sacrum (S1) is co-linear with the iliac crest. Therefore, the sacrum is not recessed within the pelvis.	Upper sacrum (S1) is caudal relative to the iliac crest. Therefore, the sacrum is recessed within the pelvis.	Pelvic outlet view
2	Mamillary processes	Transverse process of L5	Pelvic outlet view
3	The upper sacral foramens are dysmorphic: larger, noncircular, misshapen, and irregular.	The upper sacral foramens are uniformly circular and smaller.	Pelvic outlet view
4–1	The alar slope is more acute (steeper) in both the coronal and sagittal planes.	The alar slope is less acute in both the coronal and sagittal planes.	Pelvic outlet view Pelvic lateral view
4–2	The sagittal alar slope does not correlate with iliac cortical density (ICD).	The sagittal alar slope correlates with the ICD.	Pelvic lateral view
5	A residual disc space between the upper two sacral segments (between S1 and S2)	Occasionally observed	Pelvic outlet view Sagittal CT image
6	“Tongue-in-groove” sacroiliac articulation	No “tongue-in-groove” and rather flatter articulation	Axial CT image

These findings of sacral dysplasia were preventing the safe passage of a trans-iliosacral screw to S1 in surgeries for sacropelvic injuries, because the obliquely shaped alar anatomy restricts the area for screw fixation [28]. The narrow and dysmorphic osseous pathway of the upper sacral alar in a dysplastic sacrum necessitates the oblique trajectory of bilateral trans-iliosacral screw fixation to the center of the upper sacrum (S1) to stabilize the broken sacroiliac complex [28].

However, screw fixation for dysplastic upper sacrum in non-traumatic SIJ fusion does not necessarily cross the sacral foramen from the sacroiliac articulation, because fusion occurs through the relatively stable but degenerative SIJ using multiple shorter screws under fluoroscopic guidance [29]. Therefore, the vertical trajectory to the sagittal plane of the sacrum can be used for screw fixation even in cases of dysplastic upper sacrum in non-traumatic SIJ fusion. The current procedure for trans-articular screw fixation for SIJ fusion should be performed under navigation, but also could be performed under C-arm fluoroscopy using the pelvic outlet, inlet, and lateral views.

The ICD identified on the lateral image of the sacrum indicates the proximal margin of the SIJ. The proximal and distal margins of the SIJ can be identified on the AP view of the sacrum or the pelvic outlet view. The anterior and posterior margins of the SIJ cannot be clearly delineated on plain radiographs of the sacrum and pelvis, but they can be identified on a CT scan. Even with a CT scan, the anterior and posterior margins of the SIJ can be identified only partially on each axial view (or they can be identified on a transparently reconstructed sagittal image of the sacropelvic complex by marking the articular surface of the SIJ) because the sacrum contains the geographically complex sacral canal and foramens, causing unclearly delineated articulating margin of the SIJ on plain radiographs [14, 30–33]. That is why a 2D sagittal image reconstructed from 3 different sections of the mid-sagittal plane, foramens, and SIJ was used to monitor screw violations in the current study. In other words, the locations of the sacral canal, foramens, and SIJ should be evaluated preoperatively using various image studies, including a CT scan, when planning trans-articular fixation of the SIJ.

Unlike surgery to stabilize unstable sacropelvic injuries, trans-iliosacral fixation for non-traumatic SIJ pain demands trans-articular fixation for SIJ fusion [5, 15, 17]. The rate of trans-articular fixation for the PS was about 100% in both groups. The rate for the DS decreased to 63% in NDS and 74% in DYS groups. The rate for the MS was 43% in NDS and 47% in DYS groups. Therefore, trans-articular fixation with three screws developed low rates of screw pass through the articular surface and high rates of extra-articular fixation for the SIJ in the MS and DS. Unintended extra-articular fixation during the trans-articular fixation procedure for SIJ fusion penetrates the bony cortex of the ilium and/or sacrum and can injure adjacent neurovascular structures and may make SIJ fusion less effective.

Two-screw fixation for trans-articular SIJ fixation in Asians may reduce iatrogenic complications (short distal screw fixation and extra-articular fixation of the middle screw). However, the effects of race and ethnicity on iatrogenic complications following trans-articular SIJ fusion were not investigated in this study.

## Conclusion

Sacral dysplasia locates the SIJ more distally than seen in non-dysplastic sacrum, which affects the entry points and screw lengths for all screws in trans-articular SIJ fusion for non-traumatic SIJ pain. Three-screw fixation risks unstable distal screw fixation and high rates of extra-articular fixation in the middle screw.

## Data Availability

The datasets used and/or analysed during the current study are available from the corresponding author on reasonable request.

## Abbreviations

DS: distal screw
DYS: dysplastic sacrum
ICC: intra-class correlation coefficient values
ICD: iliac cortical density
MS: middle screw
NDS: non-dysplastic sacrum
PS: proximal screw
